# Unique Association between Global DNA Hypomethylation and Chromosomal Alterations in Human Hepatocellular Carcinoma

**DOI:** 10.1371/journal.pone.0072312

**Published:** 2013-09-02

**Authors:** Naoshi Nishida, Masatoshi Kudo, Takafumi Nishimura, Tadaaki Arizumi, Masahiro Takita, Satoshi Kitai, Norihisa Yada, Satoru Hagiwara, Tatsuo Inoue, Yasunori Minami, Kazuomi Ueshima, Toshiharu Sakurai, Naosuke Yokomichi, Takeshi Nagasaka, Ajay Goel

**Affiliations:** 1 Department of Gastroenterology and Hepatology, Kinki University Faculty of Medicine, Osaka-sayama, Japan; 2 Department of Gastroenterology and Hepatology, Kyoto University Graduate School of Medicine, Kyoto, Japan; 3 Outpatient Oncology Unit, Kyoto University Hospital, Kyoto, Japan; 4 Department of Gastroenterological Surgery and Surgical Oncology, Okayama University Graduate School of Medicine, Dentistry, and Pharmaceutical Sciences, Okayama, Japan; 5 Division of Gastroenterology, Department of Internal Medicine and Charles A. Sammons Cancer Center and Baylor Research Institute, Baylor University Medical Centre, Dallas, Texas, United States of America; University of Hong Kong, Hong Kong

## Abstract

Global DNA hypomethylation is a characteristic feature of cancer cells that closely associates with chromosomal instability (CIN). However, the association between these characteristics during hepatocarcinogenesis remains unclear. Herein, we determined the relationship between hypomethylation and CIN in human hepatocellular carcinoma (HCC) by analyzing 179 HCCs, 178 matched non-tumor livers and 23 normal liver tissues. Hypomethylation at three different repetitive DNA (rDNA) sequences and hypermethylation of 12 CpG loci, including 11 tumor suppressor gene (TSG) promoters, were quantified using MethyLight or combined bisulfite restriction analysis. Fractional allelic loss (FAL) was used as a marker for CIN, calculated by analyzing 400 microsatellite markers. Gains and losses at each chromosome were also determined using semi-quantitative microsatellite analysis. The associations between rDNA hypomethylation and FAL, as well as between TSG hypermethylation and FAL were investigated. Significantly more hypomethylation was observed in HCC tissues than in normal liver samples. Progression of hypomethylation during carcinogenesis was more prominent in hepatitis C virus (HCV)-negative cases, which was in contrast to our previous reports of significantly increased TSG methylation levels in HCV-positive tumors. Absence of liver cirrhosis and higher FAL scores were identified as independent contributors to significant hypomethylation of rDNA in HCC. Among the chromosomal alterations frequently observed in HCC, loss of 8p, which was unique in the earliest stages of hepatocarcinogenesis, was significantly associated with hypomethylation of rDNA by multivariable analysis (*p* = 0.0153). rDNA hypomethylation was also associated with a high FAL score regardless of tumor differentiation (*p = *0.0011, well-differentiated; *p = *0.0089, moderately/poorly-differentiated HCCs). We conclude that DNA hypomethylation is an important cause of CIN in the earliest step of HCC, especially in a background of non-cirrhotic liver.

## Introduction

Several reports suggest that promoter hypermethylation accounts for inactivation of the corresponding tumor suppressor genes (TSGs) [1]. In contrast, global DNA hypomethylation commonly found in cancer is thought to induce activation of potential oncogenes as well as chromosomal alterations, thereby contributing to carcinogenesis [2], [3]. A significant link between global DNA hypomethylation and chromosomal aberrations has been reported in several cancers, implying that global hypomethylation may play an important role in inducing chromosomal instability (CIN) [4]–[6]. Furthermore, high levels of CpG island methylation are inversely correlated with CIN in CRC, again indicating an important role for these processes in carcinogenesis [7].

In human hepatocellular carcinoma (HCC), multiple genomic alterations are thought to be involved in carcinogenesis, suggesting the heterogeneity in the molecular pathogenesis of HCC [8]. Previously, we reported that inactivation of TSGs by regional hypermethylation in their promoters is a major mechanism driving human hepatocarcinogenesis, especially in hepatitis C virus (HCV)-related cases [9]. Nonetheless, the role of increased DNA hypomethylation within different types of repetitive elements in HCC is unclear. Our goal was to determine whether DNA hypomethylation is linked to CIN and influenced by background disease and hepatitis virus infections, and if so, whether this association changes at various steps of human HCC.

In this study, we quantified DNA methylation levels at three repetitive DNA (rDNA) sequences, in the promoters of several TSGs and also determined the degree of CIN in a large number of HCC and liver tissues at various stages of tumorigenesis. Potential relationships between the degree of CIN and methylation status at rDNA and TSGs were extensively examined. We also analyzed characteristics of HCC with significant levels of DNA hypomethylation particularly in the context of degree of CIN. Our results demonstrated that global DNA hypomethylation took place at early stage of hepatocarcinogenesis especially in cases without HCV. Hypomethylation was also associated with degree of CIN and non-cirrhotic background liver. This study allowed us to provide a novel insight into the importance of epigenetic events, which may potentially drive CIN, leading to a more aggressive HCC phenotype.

## Materials and Methods

### Ethics

This study was approved by the institutional review boards of the involved institutions (reference number G365 by Kyoto University Graduate School and Faculty of Medicine, Ethnic Committee on July 13, 2010, reference number 24-001 at Kinki University Faculty of Medicine, Ethnic Committee on Apr. 20, 2012). Written informed consent was obtained from all patients.

### Samples

DNA from 179 HCCs was used for quantification of methylation levels on rDNA sequences. Among them, 66 were well-differentiated and 113 were moderately- or poorly-differentiated HCCs. Adjacent non-cancerous liver and 23 normal liver tissue samples were included [10]. Patient characteristics and distributions of tumor stages are summarized in **Table 1**. The 149 tumors and their surrounding non-cancerous liver were fresh frozen tissues. The tumors and their surrounding non-cancerous liver were frozen immediately after surgical removal and stored at −80°C until DNA isolation [11]. The remaining 30 pairs of HCC and non-cancerous liver tissues and 23 normal liver tissues were obtained as formalin-fixed paraffin-embedded samples [11]. Differentiation of HCC was determined by histological examination. Similarly, presence of liver cirrhosis (LC) was examined histologically using Ishak fibrosis score [12]. All the samples were obtained during the surgery and samples with the availability of adequate DNA quantity were selected for further analyses. Among 23 normal liver tissues, 19 specimens came from patients who had colon cancer with hepatic metastasis. The remaining normal liver tissues were from focal nodular hyperplasia, hepatic hemangioma, and hepatic adenoma [9]. Histology of normal livers showed no evidence of fibrosis or inflammation. In addition, all cases of normal liver were confirmed to be free of serum Hepatitis B virus (HBV) surface antigen and HCV antibody and to have normal serum alanine aminotransferase levels and normal blood platelet counts.

**Table 1:** Profile of patients with well-differentiated and moderately or poorly differentiated HCC.

Clinical background	HCC cases		*p* value
	Well differentiated	Moderately/poorly differentiated	
	(*n* = 66)	(*n* = 113)	
Age (y.o.)			
Median (25^th^–75^th^ percentiles)	61 (56–68)	60 (54–66)	0.3817*
**Gender**			
male/female/missing data	44/22/0	75/35/3	0.8353†
Hepatitis virus			
B/BC/C/NBNC‡	12/0/51/3	27/3/64/19	0.0166†
Adjacent non-cancerous liver			
non-LC/LC/missing data§	23/42/1	32/74/7	0.4801†
Child-Pugh classification			
Grade A/Grade B/missing	57/5/4	54/5/54	0.9347
Tumor size (cm)			
Median (25^th^–75^th^ percentiles)	2.5 (1.4–4.0)	3.2 (2.5–6.0)	0.0021*
Serum AFP levels (ng/ml)			
Median (25^th^–75^th^ percentiles)	16.9 (5.6–55.4)	119 (14.7–1615.8)	0.0009*

CI, confidence interval; HCC, hepatocellular carcinoma;

* *p* value by Wilcoxon rank-sum test;

† *p* value by the chi-square test. Missing cases of gender, missing adjacent non-cancerous liver samples, or hepatitis virus-positive cases carrying both HBs Ag and HCV Ab were excluded from statistical analysis using the chi-square test.

‡ “B” denotes the cases with HBsAg-positive, “BC” denotes both HBsAg and HCV Ab-positive, “C” denotes HCV Ab-positive, and “NBNC” denotes the cases with both negative, respectively. All non-cancerous liver of HBV or HCV-positive cases was revealed as chronic hepatitis or liver cirrhosis according to the histological examination.

§ “non-LC” denotes background liver without cirrhosis and “LC” denotes liver cirrhosis. The presence of LC was determined by histological examination [12]. Child-Pugh classification represented the background liver function.

### Quantification of Methylation Levels in Repetitive DNA Sequences and TSG Promoters

DNA extraction and bisulfite modification treatment were described previously [13]. For extraction of tumorous DNA, we carefully selected tumorous tissue without containing non-tumorous surrounding liver. rDNA methylation levels were quantified at two types of interspersed nucleotide repeats, long interspersed nuclear element-1 (LINE-1) and Alu, and one juxtacentromeric heterochromatin region, juxtacentromeric satellite 2 (SAT2), using the MethyLight methodology. The analysis of Alu sequences was performed using the consensus Alu sequence, and details of all PCR primers and probes used in this assay have been described previously [14]. Real-time quantitative PCR was performed using a StepOne real-time detection system (Applied Biosystems, Foster City, CA). PCR was performed according to the manufacturer’s protocol using TaqMan Fast universal PCR Master Mix (Applied Biosystems). A standard curve for each assay was generated from serial dilutions of the reference sample, bisulfite-treated CpGenome Universal Methylated DNA (CHEMICON International Inc., Temecula, CA). The methylation-independent consensus Alu sequence was used as an endogenous control, as described previously [14]. Methylation levels at each rDNA sequence were normalized to those of CpG methylase-treated DNA.

Quantification of methylation levels in 11 TSG promoters and 1 MINT locus (*APC*, *CACNA1G*, *CASP8*, *CDKN2A*, *GSTP1*, *HIC1*, *PRDM2*, *PTGS2*, *RASSF1*, *RUNX3*, *SOCS1*, and MINT31) was performed using combined bisulfite restriction analysis, as previously described [9]. Based on our previous study, we selected 12 CpG loci for evaluation of status of regional hyermethylation because methylation levels of these CpG loci were markedly higher in well-differentiated HCC compared to non-cancerous liver, suggesting their potential role in early steps of human hepatocarcinogenesis [10].

### Classification of HCC According to the Methylation Levels in Repetitive DNA Sequences and TSG Promoters

We applied hierarchical clustering analysis using methylation levels of 3 rDNA sequences as well as those of 11 TSG promoters and 1 MINT locus to discriminate tumors according to degree of hypomethylation and hypermethylation, respectively, because hierarchical clustering analysis is the most appropriate method to statistically discriminate HCC according to methylation levels of multiple loci. We compared the methylation levels of each cluster and classified HCCs as having either significant hypomethylation or slight hypomethylation in rDNA sequences and with either extensive hypermethylation or limited hypermethylation at the 12 CpG loci of the TSG promoters/MINT locus [9].

### Quantification of Chromosomal Alterations by Fractional Allelic Loss

In order to determine the amount of chromosomal alterations in HCC samples, we analyzed allelic imbalance (AI) in 110 out of 179 liver tumor samples using 400 microsatellite markers equally distributed throughout all 23 chromosomes (ABI PRISM Linkage Mapping Set MD-10, Applied Biosystems). We could not obtain enough DNA from the remaining 69 samples for this analysis. Details of PCR conditions and assessment of AI were published previously [15]. Fractional allelic loss (FAL) scores, which broadly represent an index of CIN, were calculated as the number of microsatellite loci with AI divided by number of total informative loci and expressed as a percentage. We also evaluated allelic dose with multiples PCR using a retained allele and determined whether AI was the results of chromosomal gain or loss as described previously [15].

### Statistical Analysis

We use Pearson’s chi-square test or Fisher’s exact test for comparison of categorical variables and Wilcoxon rank-sum test and Student’s *t*-test for continuous variables. In order to compare the amount of methylation in all rDNA sequences (used to indicate global DNA hypomethylation) among different stages of liver tissues, analysis of variance (ANOVA) with post-hoc Tukey-Kramer honestly significant difference (HSD) multiple comparison was applied. For normalization of methylation levels on multiple loci, the *Z* score was applied which was defined as difference between individual and mean methylation level divided by standard deviation [9]. The mean and median value of FAL was 20%. Therefore, to discriminate HCCs according to the degree of CIN, we also classified tumors into two groups: those with an FAL score >20% and those with an FAL score ≤20%. To identify independent predictors of significant hypomethylation, we used multiple logistic regression analysis. All *p* values were two-sided, and *p*<0.05 was considered to indicate statistical significance. All statistical analyses were conducted using the JMP version 9.0 software (SAS Institute Inc., Cary, NC).

## Results

### Hypomethylation Status of rDNA Elements at Different Steps of Hepatocarcinogenesis

We compared the methylation levels of three different rDNA sequences in normal liver, non-tumor liver from HCC patients, and well-differentiated or moderately/poorly-differentiated HCC tissues [16]. Profiles of patients with tumors of each differentiation are shown in **Table 1**. HCV-positive status was more frequent in HCC samples classified as well-differentiated (*p = *0.0116; chi-square test). Similarly, patients with well-differentiated HCC had smaller tumors and lower serum alpha-fetoprotein (AFP) levels (*p = *0.0021 and *p = *0.0009, respectively; Wilcoxon rank-sum test). For analysis of progression of hypomethylation during early step of hepatocarcinogenesis, we also compared rDNA methylation levels in well-differentiated HCCs of two different size categories: ≤2.0 cm and >2.0 cm.

**Figure 1:**
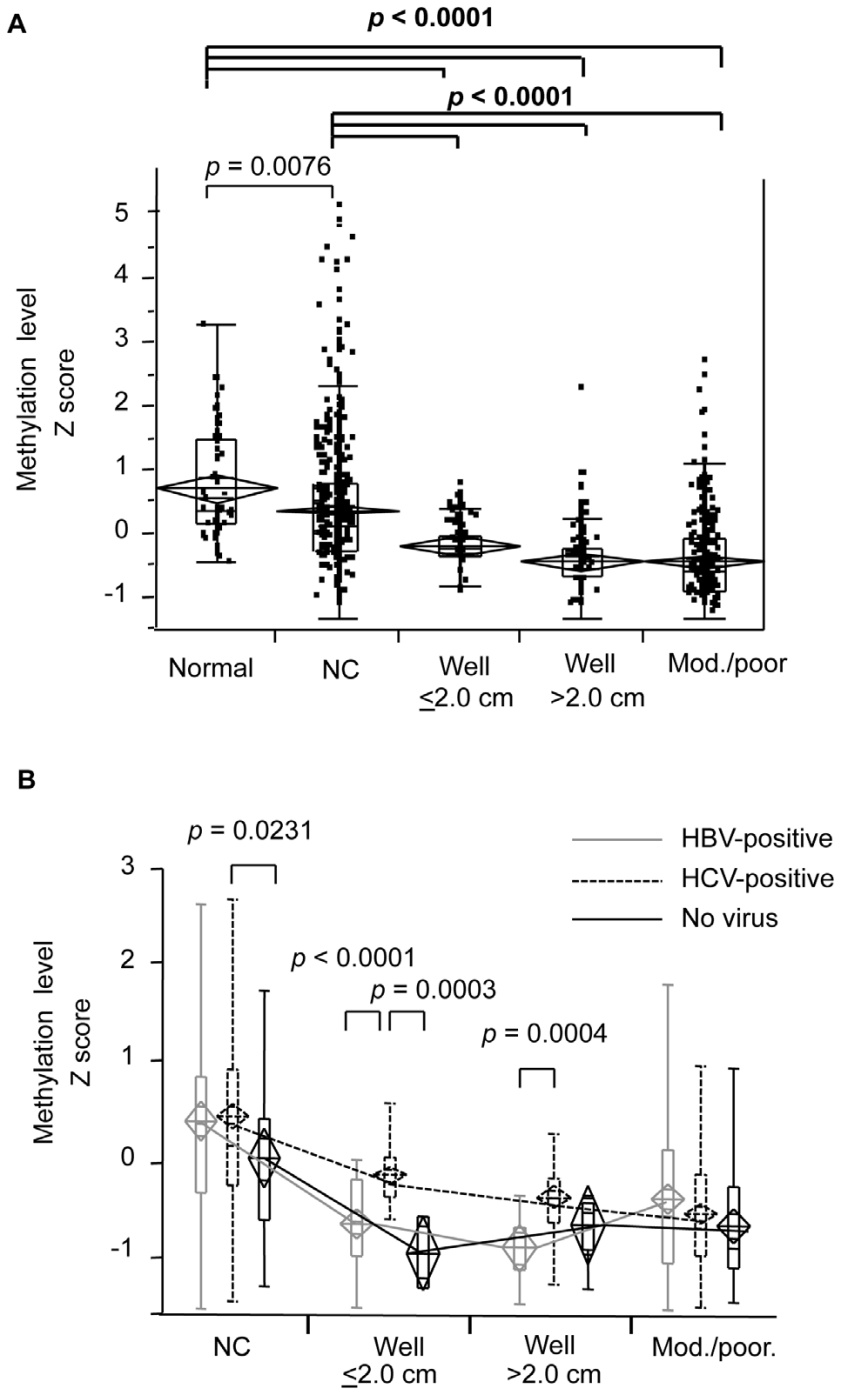
Distribution of the percentage methylation levels of repetitive DNA sequences in liver tissues. (A) Distribution of the percentage methylation levels (shown as *Z* scores) in all tumors. Box and whisker plots denote 75% and 95% distributions; lines within boxes show median values; mean methylation levels and 95% CI are shown as diamonds and lines within the diamonds, respectively. ‘Normal’ denotes normal liver (*n* = 69); ‘NC’ denotes matched, non-cancerous liver samples (*n* = 520); ‘Well’ denotes well-differentiated HCCs (*n* = 87 for ≤2.0 cm; *n* = 111 for >2.0 cm); Mod./poor denotes moderately or poorly differentiated HCCs (*n* = 339). *P* values were calculated by post-hoc Tukey-Kramer HSD multiple comparison. Significant differences (*p*<0.0001) are shown in bolt lines. The F and *p* values for the ANOVA test are as follows: F (4, 1125) = 69.64; *p*<0.0001. (B) Significant hypomethylation of repetitive DNA sequences in tumors from HBV-positive (gray solid line), HCV-positive (black dashed line), and virus-negative (black solid line) patients. Methylation levels of three sequences (Alu, LINE-1, and SAT2) in each type of liver tissue were normalized to CpG methylase-treated DNA levels and expressed as a *Z* score. The box and whisker plots denote 75% and 95% distributions; lines within boxes show median values; mean methylation levels and 95% CI are shown as diamonds and lines within the diamonds, respectively. The three samples carrying both HBV and HCV were excluded from this analysis. The greatest difference in HCC hypomethylation was between virus-negative and HCV-infected tumors. *P* values were calculated using Tukey-Kramer HSD multiple comparison. The *p* values for each ANOVA test are as follows: F (2, 485) = 3.50, *p* = 0.0311 for NC; F (2, 78) = 22.21, *p*<0.0001 for well-differentiated HCCs≤2.0 cm; F (2, 105) = 8.32, *p* = 0.0004 for well-differentiated HCCs >2.0 cm.

Of the CpG loci analyzed, methylation at the Alu and SAT2 sequences in non-cancerous liver tissues was slightly lower than that in normal liver (*p* = 0.0494 for Alu and *p* = 0.0334 for SAT2: **Fig. S1**). HCC tumors at all stages of development had less rDNA methylation compared to normal liver and non-cancerous liver tissue at all three sequences, suggesting that hypomethylation is specific to carcinogenesis (**Table 2** and **Fig. S1)**. **Fig. 1A** shows the decrease in global methylation levels at different HCC stages expressed as a *Z* score of methylation levels at all three rDNA elements. Overall, rDNA methylation decreased with progression of the liver disease (*p*<0.0001, ANOVA: **Fig. 1A**). Less methylation was observed in non-cancerous liver tissues compared to normal liver tissues (*p* = 0.0076, post-hoc Turkey-Kramer HSD multiple comparison), and methylation levels in HCC tissues were significantly decreased compared to normal liver samples even at the earliest stages of tumor development (*p*<0.0001: **Fig. 1A**).

**Table 2:** Mean methylation levels in different liver tissues.

Locus		Mean methylation level (%; 95% CI)				
	Normal liver	Non-cancerous liver	Well-differentiated HCC		Moderately or poorly-differentiated HCC	*p* value according to ANOVA*
			≤2.0 cm	>2.0 cm		
	(*n* = 23)	(*n* = 178)	(*n* = 29)	(*n* = 37)	(*n* = 113)	
Alu	0.70 (0.62–0.78)	**0.58 (0.55–0.61)**	**0.46 (0.41–0.50)**	**0.39 (0.35–0.42)**	**0.41 (0.37–0.45)**	**<0.0001**
LINE-1	0.72 (0.63–0.82)	0.69 (0.65–0.74)	**0.49 (0.42–0.56)**	**0.35 (0.29–0.41)**	**0.35 (0.31–0.38)**	**<0.0001**
SAT2	1.33 (1.14–1.53)	**1.02 (0.93–1.12)**	**0.54 (0.48–0.60)**	**0.50 (0.40–0.60)**	**0.45 (0.38–0.52)**	**<0.0001**

CI, confidence interval; HCC, hepatocellular carcinoma. Mean percent methylation (95% CI) at individual CpG loci in each type of liver sample is shown. Values in bold denote significant differences in methylation levels compared to normal liver tissue.

* F values are as follows: F (4, 374) = 21.87 for Alu; F (4, 375) = 50.78 for LINE1; F (4, 374) = 37.00 for SAT2.

### Hypomethylation Reduces during HCC Development with Differing Viral Status

Next, we wanted to determine whether viral status affected alterations in rDNA methylation during tumor progression. We compared methylation levels in rDNA sequences from HBV, HCV, and virus-negative human HCC tumor samples. Hypomethylation of rDNA sequences was more prominent in HCV-negative tumors than in HCV-infected tumors in well-differentiated HCCs, especially in tumors <2.0 cm (well-differentiated HCCs≤2.0 cm in size: *p*<0.0001 for HBV-infected vs. HCV-infected tumors, *p* = 0.0003 for virus-negative vs. HCV-infected tumors; well-differentiated HCC≥2.0 cm: *p* = 0.0004 for HBV-infected vs. HCV-infected tumors; post-hoc Tukey-Kramer HSD multiple comparison; **Fig. 1B**). However, there were no significant differences associated with viral status among moderately or poorly differentiated HCCs. Thus, these data indicate that an increase in rDNA demethylation occurs more profoundly during early-stage tumor development in HCV-negative than in HCV-positive hepatocarcinogenesis.

### Characterization of HCCs with Significant DNA Hypomethylation

**Figure 2:**
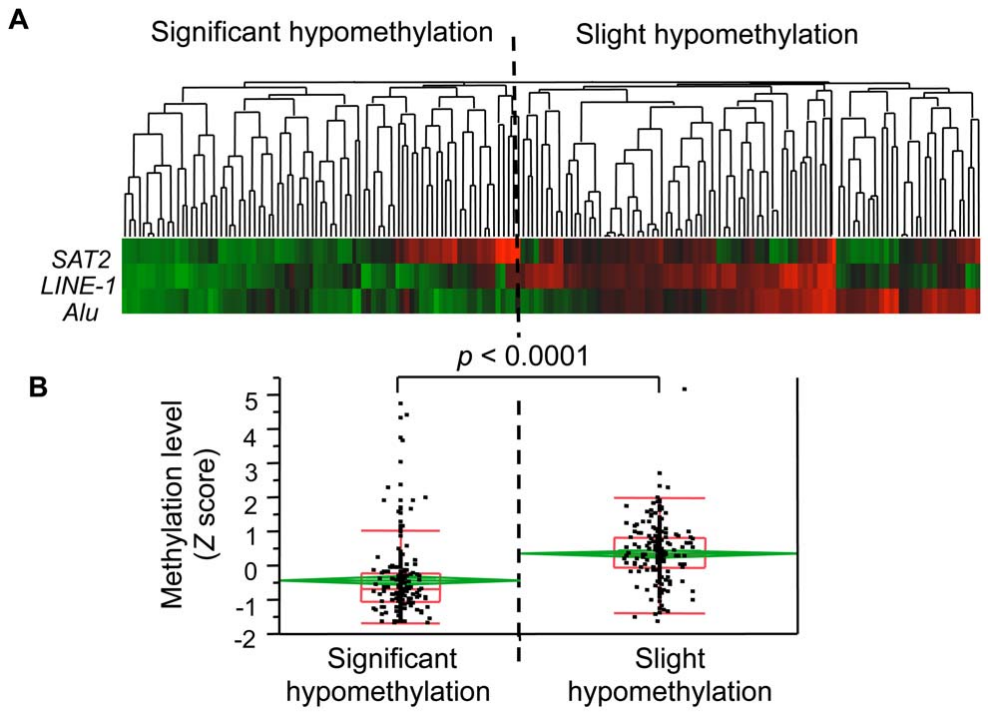
Categorization of tumors with significant hypomethylation of repetitive DNA sequences. All tumors were classified as either having “significant” or “slight” hypomethylation based on the methylation signatures at three different repetitive DNA elements (Alu, LINE-1 and SAT2). (A) The color map represents: green, low methylation level; red, high methylation level. (B) Distribution of methylation levels for each tumor subgroup classified in (A). Box and whisker plots (red line) denote 75% and 95% distribution; lines in the boxes denote median values; diamonds and lines within diamonds (green line) indicate the mean and 95% CI values, respectively. Methylation levels are expressed as *Z* scores. Mean and median *Z* values were −0.45949 and −0.66030 for HCCs with significant hypomethylation; and 0.34908 and 0.32342 for tumors with slight hypomethylation. *P* values were determined using the Student’s *t*-test and the Wilcoxon rank-sum test. Both tests yielded the same *p* value.

We classified HCCs with significant global DNA hypomethylation according to methylation levels at Alu, LINE-1, and SAT2 sequences by using hierarchical clustering analysis, which objectively identifies statistical differences in DNA demethylation profiles (**Fig. 2A** **)**. Following this analysis, all 179 HCCs were classified into two subclasses: in 83 liver tumors with significant levels of hypomethylation, rDNA methylation was much lower than that in HCCs with slight hypomethylation (mean and median *Z* scores were −0.45949 and −0.66030 for samples with significant hypomethylation versus 0.34908 and 0.32342 for those with slight hypomethylation, *p*<0.0001; Student’s *t*-test and Wilcoxon rank-sum test; **Fig. 2B**). These results indicate that significant differences in global DNA hypomethylation levels in HCC tumors classified as having significant hypomethylation compared with those with slight hypomethylation. We also compared each methylation levels of Alu, LINE-1 and SAT2 between tumors classified as significant hypomethylation and slight hypomethylation. Methylation levels of tumors with significant hypomethylation were markedly lower than those of slight hypomethylation for all 3 CpG loci. These results also conformed that the classification by hierarchal clustering analysis is appropriate to discriminate the tumors based on global hypomethylation (*p*<0.0001 for Alu, *p*<0.0001 for LINE-1, and *p* = 0.0094 for SAT2 by Wilcoxon rank-sum test; **Table S1)**.

Variables such as age (>60 y.o., *p* = 0.0292), gender (male, *p = *0.0440), viral status (non-HCV, *p = *0.0337), status of normal adjacent liver (non-LC, *p = *0.0001), tumor size (>2.0 cm, *p*<0.0001), tumor differentiation (moderately or poorly differentiated, *p* = 0.0075), and FAL score (>20%, *p* = 0.0079) were all associated with significant hypomethylation (**Table S2)**. When we compared FAL scores as continuous variables in tumors with significant versus slight hypomethylation, tumors with significant hypomethylation had higher FAL scores (mean and median FAL scores of 27.1% vs. 18.5% and 23.1% vs. 17.0%, respectively, for HCCs with significant hypomethylation vs. slight hypomethylation; *p* = 0.0012, Student’s *t*-test, and *p = *0.0023, Wilcoxon rank-sum test: **Table S2**).

**Figure 3:**
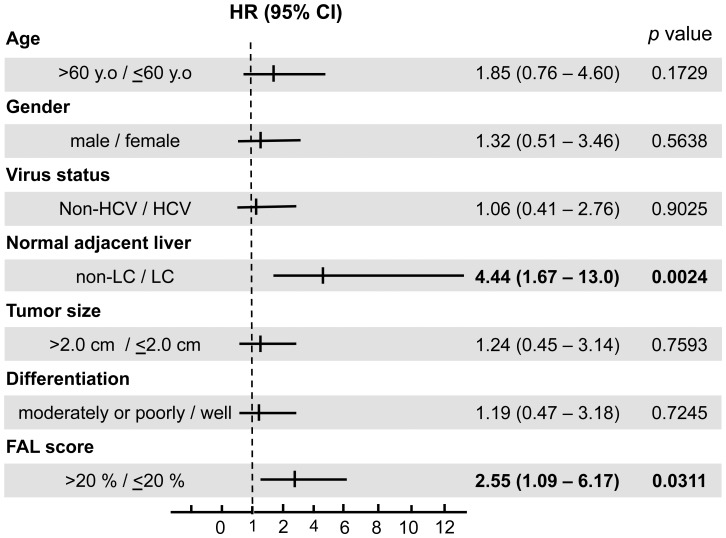
Multivariate analysis for contribution of each variable to significant hypomethylation in HCC. The *p* values were calculated using multiple logistic regression analysis. The total number of patients and the number of cases with significant hypomethylation in each group and the associated *p* values of univariate analyses calculated using the chi-square test are shown in **Table S2**. *P* values <0.05 are shown in bold.

To further analyze the contribution of each variable to hypomethylation levels in HCC, we applied multiple logistic regression analysis. Among the variables which showed significant relation to hypomethylation, non-LC and higher FAL score were identified as independent contributors to significant global hypomethylation (*p* = 0.0024, odds ratio = 4.44, 95% CI = 1.67–13.0 for non-LC; *p* = 0.0311, odds ratio = 2.55, 95% CI = 1.09–6.17 for FAL score >20%; **Fig. 3**).

### Association between Alterations on Specific Chromosomal Arms and rDNA Hypomethylation

**Figure 4:**
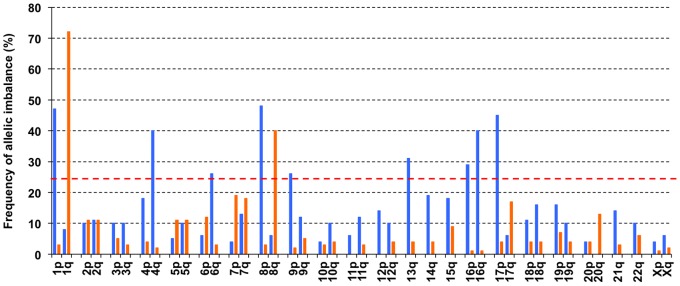
Frequencies of allelic imbalance in HCC on each chromosomal arm. The vertical bars show frequencies of allelic loss (shown in blue) and allelic gain (red); the allelic doses were determined by multiples PCR using a retained allele as a internal control [14]. The red horizontal dotted bar indicates the frequencies of 25%.

According to semi-quantitative microsatellite analyses, the following chromosomal arms showed frequent alterations at more than 25% of tumors, which is a unique observation in human HCC: loss of 1p (45%), 4q (42%), 6q (28%), 8p (54%), 9p (28%), 13q (34%), 16p (30%), 16q (41%), and 17p (48%); gain of 1q (71%) and 8q (42%) (**Fig. 4**). Among these, we tried to clarify chromosomal alterations specifically affected by global hypomethylation. For this purpose, we compared frequencies of losses and gains of these chromosomal arms between tumors with significant and slight hypomethylation (**Table 3**). Of these, loss of 6q, 8p, 13q, and 17p were significantly associated with significant global hypomethylation. Notably, non-LC and loss of 8p was also identified as independent factors for accompanying significant global hypomethylation by multivariable analysis using age, gender, virus status, tumor size, tumor differentiation and loss of 6q, 8p, 13q, and 17p as co-variables (*p* = 0.0018, odds ratio = 5.19, 95% CI = 1.81–16.2 for non-LC; *p* = 0.0153, odds ratio = 3.14, 95% CI = 1.24–8.28 for loss of 8p: **Table 4**).

**Table 3:** Association between significant hypomethylation on repetitive DNA and alteration of specific chromosomal arms.

chromosome	Number of cases with significant hypomethylation/total cases (%)	*P* value	chromosome	Number of cases with significant hypomethylation/total cases (%)	*P* value
Loss of 1p			Loss of 9p		
with	20/54 (37%)		with	15/30 (50%)	
without	30/59 (51%)	0.1398	without	35/83 (42%)	0.4592
Gain of 1q			**Loss of 13q**		
with	34/82 (41%)		**with**	**21/35 (60%)**	
without	16/31 (52%)	0.3324	**without**	**29/78 (37%)**	**0.0239**
Loss of 4q			Loss of 16p		
with	23/46 (50%)		with	16/33 (48%)	
without	27/67 (40%)	0.3077	without	34/80 (43%)	0.5603
**Loss of 6q**			Loss of 16q		
** with**	**18/28 (64%)**		with	21/46 (46%)	
** without**	**32/85 (38%)**	**0.0138**	without	29/67 (43%)	0.8033
**Loss of 8p**			**Loss of 17p**		
** with**	**33/55 (60%)**		**with**	**28/51 (55%)**	
** Without**	**17/58 (29%)**	**0.0010**	**without**	**22/62 (35%)**	**0.0386**
Gain of 8q					
with	23/46 (50%)				
without	27/67 (40%)	0.3077			

Among 11 chromosomal arms frequently altered in HCC, significant association between alteration of chromosomal arms and global hypomethylation (determined by methylation status of repetitive DNA) were observed for loss of 6q, 8p, 13q, and 17p (shown in bold). Each *p* value was determined by chi-square test.

**Table 4:** Multivariate analysis for the contribution of specific chromosomal alterations to significant hypomethylation in HCC.

	Univariate analysis	Multivariate analysis	
Variables	*p* value*	*p* value†	Odds ratio (95% CI)
Age (y.o)			
≤60	–	–	1
>60	**0.0292**	0.3329	1.61 (0.61–4.26)
Gender			
Female	–	–	1
Male	**0.0440**	0.4441	1.48 (0.55–4.12)
Virus status			
HCV	–	–	1
Non-HCV	**0.0337**	0.2106	1.93 (0.69–5.70)
Normal adjacent liver			
LC	–	–	1
Non-LC	**0.0001**	**0.0018**	**5.16 (1.81–16.2)**
Tumor size			
≤2.0 cm	–	–	1
>2.0 cm	**<0.0001**	0.9303	1.06 (0.27–4.61)
Differentiation			
Well	–	–	1
Moderately/Poorly	**0.0075**	0.9000	1.08 (0.34–3.53)
Loss of 6q			
Absent	–	–	1
Present	**0.0428**	0.1592	5.27 (0.55–128)
Loss of 8p			
Absent	–	–	1
Present	**0.0010**	**0.0153**	**3.14 (1.24–8.28)**
Loss of 13q			
Absent	–	–	1
Present	**0.0239**	0.4176	1.54 (0.54–4.47)
Loss of 17p			
Absent	–	–	1
Present	**0.0386**	0.0850	2.42 (0.89–6.95)

HCC, hepatocellular carcinoma;

* *p* value from the chi-square test or Fisher’s exact test for comparison of two categorical variables;

† *p* value from multiple logistic regression analysis; *p* values <0.05 are shown in bold. Numbers of each case were shown in **Table S2** and **Table 3**.

### rDNA Hypomethylation is Associated with Chromosomal Instability in HCC

Multivariate analysis revealed that a high FAL score is an independent factor related to significant hypomethylation. To confirm that the association between significant hypomethylation and FAL score is tumor stage-independent, FAL scores were analyzed by hypomethylation status using hierarchical clustering analyses (significant vs. slight) within each tumor grade (well-differentiated and moderately/poorly differentiated). We performed a similar analysis on tumor grade categorized according to extensive or limited TSG hypermethylation by using hierarchical clustering analysis of methylation levels at the 12 selected TSGs/MINT loci. The classification of significant or slight global hypomethylation, and extensive or limited TSG hypermethylation within each tumor grade based upon hierarchical clustering analyses is shown in **Figure S2**.

**Figure 5:**
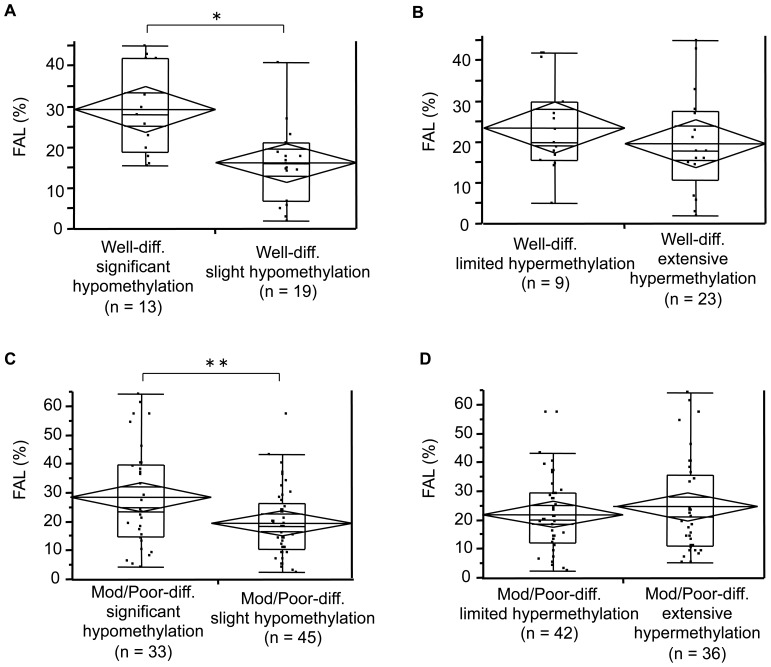
Association of FAL score and methylation status of repetitive DNA and TSG promoter in HCCs. Comparisons of FAL score (%) between HCCs with significant or slight hypomethylation at repetitive DNA (A, C), and between tumors with extensive or limited hypermethylation at TSG promoter (B, D) in well-differentiated and moderately/poorly differentiated HCCs. ‘Well-diff.’ denotes well-differentiated tumors and ‘Mod/Poor diff.’ denotes moderately/poorly differentiated tumors. Tumors were classified as having significant or slight hypomethylation following hierarchical clustering analysis of methylation levels at three repetitive DNA sequences (Alu, LINE-1 and SAT2: **Figure S2A, S2C**). Similarly, tumors were classified as having extensive or limited TSG hypermethylation following hierarchical clustering analysis of methylation levels at 12 TSGs/MINT loci that are frequently involved in HCC (**Figure S2B, S2D**). n = number of FAL scores in each group. Box and whisker plots denote 75% and 95% distribution; lines in the boxes denote median values; diamonds and lines within the diamonds indicate the mean and 95% CIs, respectively. *, *p* = 0.0011 by Student’s *t*-test and *p* = 0.0015 by Wilcoxon rank-sum test; **, *p* = 0.0089 by Student’s *t*-test and *p* = 0.0270 by Wilcoxon rank-sum test.

As shown in **Fig. 5**, higher FAL scores were exclusively associated with significant hypomethylation in well-differentiated tumors (*p = *0.0011, Student’s *t*-test; *p = *0.0015, Wilcoxon rank-sum test; **Fig. 5A** **)**, but showed no association with extensive TSG methylation (*p = *0.2670, Student’s *t*-test; *p = *0.1601, Wilcoxon rank-sum test; **Fig. 5B**). An association between global hypomethylation and CIN phenotype was also observed in moderately or poorly differentiated HCCs; HCCs with significant hypomethylation carried higher FAL scores than those with slight hypomethylation (*p* = 0.0089, Student’s *t*-test; *p = *0.0270, Wilcoxon rank-sum test; **Fig. 5C** **).** Again, no association was observed between TSG methylation levels and FAL scores (*p = *0.4527, Student’s *t*-test; *p* = 0.6663, Wilcoxon rank-sum test; **Fig. 5D**). Since we used matched pairs of tissue samples of HCC and non-cancerous liver, we also calculated the differences in the methylation levels between non-cancerous liver and HCC, and examined the relationship between the difference of methylation levels and FAL scores. The median difference of Z scores was 0.3; therefore we arbitrarily classified cases as with progressive hypomethylation if a difference in Z score was 0.3 or more. The cases with progressive hypomethylation carried HCC with higher FAL scores compared to those without progressive hypomethylation (*p* = 0.0040 by student-t test and *p* = 0.0056 by Wilcoxon rank-sum test: **Table S3**).

## Discussion

In this study, we quantitatively and comprehensively analyzed DNA hypomethylation and CIN in HCCs using a structured approach that involved analysis of liver tissues during several stages of HCC development. Our findings indicated that rDNA hypomethylation increases with progression of liver disease. However, according to the analyses of rDNA hypomethylation and chromosomal alterations, hypomethylation is clearly associated with the amount of chromosomal alterations, regardless of tumor differentiation status. In addition, significant global hypomethylation is more often observed in non-cirrhotic livers; well-differentiated HCCs that are HCV-negative show greater hypomethylation than HCV-positive HCCs.

Repetitive DNA elements comprise approximately 45% of the human genome and consist of interspersed repeats and tandem repeats of simple (satellite DNA) or complex sequences. The Alu repeat and the LINEs are abundant nucleotide elements; their methylation status is reported to be associated with global methylation levels [14]. In contrast, satellite DNA is largely confined to centromeres or juxtacentromeric chromatin, and SAT2 is predominantly found in the juxtacentromeric heterochromatin of specific human chromosomes, such as chromosomes 1 and 16, where chromosomal alterations are frequently reported in HCC [17]. Therefore, we consider that methylation levels at these three types of rDNA sequences are representative of the global DNA methylation status [14], [18]. Secondly, we investigated changes in hypomethylation at defined stages of HCC development. Several reports suggested that increases in global DNA hypomethylation are related to advanced tumor stages with poor tumor differentiation and argued that this phenomenon might be a consequence of carcinogenesis [6], [19]. However, our analysis showed that significant demethylation could be detected in HCC regardless of tumor differentiation, compared to normal liver and adjacent non-cancerous tissue from HCC patients, suggesting an important role of global hypomethylation on emergence of HCC. However, despite the histological and serological determination of normal liver in this analysis, samples as normal controls were obtained from patients with metastatic colon cancer, which might affect the global methylation status of the liver. As we could not rule out the use of chemotherapy before liver resection, this might affect the methylation status in normal liver samples. In addition, selection of the patients may be affected by the liver function, as only patients with good liver function would get surgical resection, which may possibly be a potential limitation of this study.

Our previous study suggested that TSG inactivation via abnormal promoter methylation is a common occurrence, especially in HCV-related HCCs [9]. In contrast, in the present study, rDNA methylation levels at the early steps of tumor development were significantly lower in HCV-negative HCCs than in HCV-positive tumors. This suggests that increased DNA hypomethylation could be a unique characteristic at early step of hepatocarcinogenesis in HCV-negative livers. To confirm these traits in HCCs carrying significant hypomethylation, we applied hierarchical clustering analysis and categorized HCCs by increasing hypomethylation based on Alu, LINE-1, and SAT2 methylation. In addition to the absence of HCV, older age (>60 y.o.), male gender, absence of cirrhosis, large tumor size, tumor dedifferentiation, and high FAL scores were also associated with significant tumor hypomethylation. Some report suggested the association between DNA hypomethylation and gender as well as aging [20], [21]. Global DNA methylation level in the mouse liver is reportedly affected by methyl-deficient diet more profoundly in male mice [20]. Aging might also affect a global DNA methylation level although it should be confirmed by a large-scale study [21]. So far, it might be attractive to speculate that DNA hypomethylation in the background liver might accelerate an emergence of HCC with significant hypomethylation, although no significant differences in the degree of hypomethylation was detected between non-LC and LC in background liver in this study (p = 0.8397 and 0.1081 by Student’s t-test and Wilcoxon rank-sum test, respectively; data not shown). On the other hand, multivariate analysis revealed that the absence of cirrhosis and high FAL scores were independent risk factors for significant hypomethylation, supporting the idea that HCC with significant global hypomethylation tend to carry high FAL and emerge from background liver without cirrhosis.

In this study, we also found a significant correlation between global DNA hypomethylation and specific chromosomal alterations: losses of 6q, 8p, 13q, and 17p. Loss of 8p was identified as an independent factor for accompanying significant hypomethylation. Interestingly, recurrent losses of 8p and 17p were reportedly observed even in well-differentiated HCC and loss of 8p was unique in the earliest stages of hepatocarcinogenesis [15]. Therefore, to confirm an association between global hypomethylation and CIN at early steps of hepatocarcinogenesis, we classified both well-differentiated and moderately/poorly differentiated tumors as having “significant” or “slight” hypomethylation using hierarchical clustering analysis and compared FAL scores between them. Interestingly, even in well-differentiated HCCs, tumors with significant levels of hypomethylation were clearly associated with high FAL scores, while no association was detected between extensive TSG hypermethylation and high FAL scores. Similar results were observed in moderately or poorly differentiated tumors. In addition, progression of hypomethylation from surrounding background liver to HCC tissues was also associated with higher FAL of HCC tissues. These results indicate that the relationship between DNA hypomethylation and high FAL scores is stage-independent and support the idea that global DNA hypomethylation is not simply a consequence of tumor progression but induces chromosome fragility, which could in turn lead to CIN in HCC, even at early steps of tumorigenesis.

Although mouse models revealed a clear association between DNA hypomethylation and the induction of CIN, the mechanism is still not clear. Activation of retrotransposition can lead to chromosomal arrangements [2], [22]. Recently, Stefanska *et al*. reported that promoter DNA hypomethylation induces the expression of several genes involved in cell growth, signal transduction, and invasion, and thus contributes to hepatocarcinogenesis [23]. Therefore, genes that are activated through promoter DNA hypomethylation may also cause CIN if such hypomethylation is associated with rDNA hypomethylation within these genes [24]. Further study is required to clarify how DNA hypomethylation could be responsible for induction of CIN during hepatocarcinogenesis.

Our analysis revealed that HCC with significant hypomethylation is characterized by both a lack of cirrhosis and high FAL scores. Liver cirrhosis is a well-recognized premalignant condition, especially in HCV-positive patients. However, HCC can develop in the absence of cirrhosis, especially in HCV-negative cases [25]. Interestingly, TSG hypermethylation is reported to be more prevalent in HCC arising in a background of liver cirrhosis [26]. Another report suggested that environmental factors such as alcohol intake and HBV infection could contribute to HCC through global hypomethylation [18], [27]. Recent analysis of whole genome sequence of HCC also revealed that a high degree of copy number alteration was more frequently observed in HBV-related tumor and tumors developed in non-cirrhotic liver [28]. Therefore, patients without HCV or cirrhosis may be more likely to develop HCC via DNA hypomethylation and CIN-related pathways, in contrast to HCV-related carcinogenesis, where HCV infection may reportedly introduce methylation-related TSG inactivation [9].

In this study, we characterized HCC cases carrying significant global DNA hypomethylation. rDNA hypomethylation occurred at an earlier step of hepatocarcinogenesis in the absence of HCV, and significant hypomethylation was associated with CIN and the absence of liver cirrhosis. This suggests that more than one pathway is involved in hepatocarcinogenesis; in the absence of HCV, increased global DNA hypomethylation is accompanied by CIN, which differs from the “CpG island methylator (CIMP) pathway” involved in HCV-related hepatocarcinogenesis [9]. Recently, DNA methyltransferase inhibitor and histone deacethylase inhibitor are applied for epithelial malignancy including HCC [29], [30]. However, as both “CIMP” and “global hypomethylation and CIN” type pathways were suspected to exist, analysis of methylation profile should be critical for management of HCC.

## Supporting Information

Figure S1 **Alterations in methylation levels of repetitive DNA sequences.**
(DOC)

Figure S2 **Hierarchical clustering analysis for categorization of methylation status of global hypomethylation and hypermethylation of TSGs in well-differentiated HCCs and moderately or poorly differentiated HCCs.**
(DOC)

Table S1 **Difference of methylation levels of Alu, LINE-1, and SAT2 between tumor with significant hypomethylation and with slight hypomethylation.**
(DOC)

Table S2 **Univariate analysis of the contribution of each variable to significant hypomethylation at repetitive DNA sequences in HCC.**
(DOC)

Table S3 **Association between progression of hypomethylation and FAL score of HCC.**
(DOC)
