# Supplementary material for: Unique Association between Global DNA Hypomethylation and Chromosomal Alterations in Human Hepatocellular Carcinoma
Source: PLoS One. 2013 Sep 2;8(9):e72312. doi: 10.1371/journal.pone.0072312 (PMC3759381; doi:10.1371/journal.pone.0072312)
Supplement: Figure S1 — Alterations in methylation levels of repetitive DNA sequences. (DOC) [file pone.0072312.s001.doc]

**Supplementary Figure S1: Alterations in methylation levels of repetitive DNA sequences.**


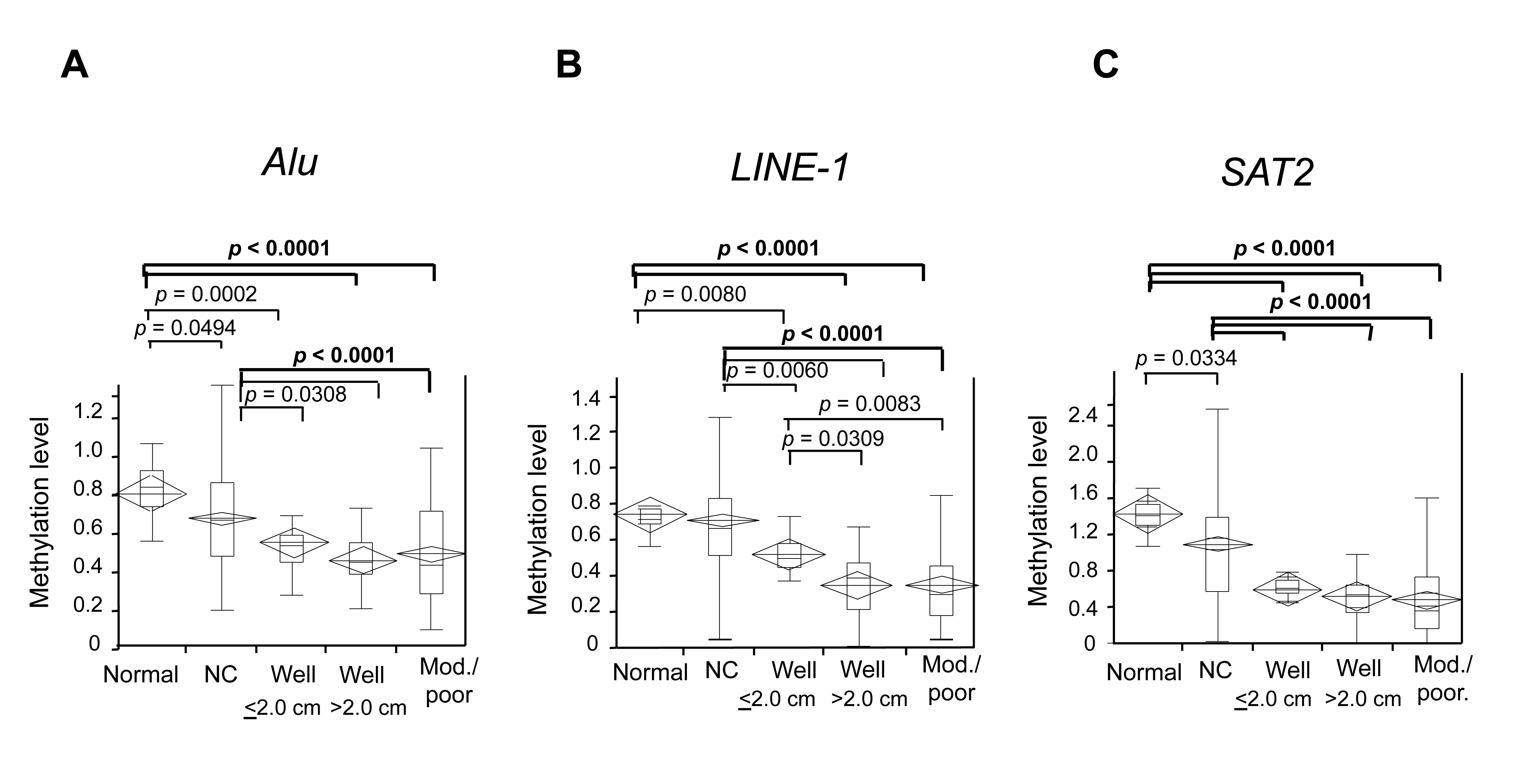


Alterations in methylation levels of repetitive DNA sequences in Alu (A), LINE-1 (B), and SAT2 (C), in normal liver, non-cancerous liver from patients with HCC, and each HCC tumor stage. Box and whisker plots denote 75% and 95% distributions; lines within boxes show median values; mean methylation levels and 95% CI are shown as diamonds and lines within the diamonds, respectively. The vertical showed methylation level of each repetitive DNA sequences which was normalized to that of CpG methylase-treated DNA. *P* values were determined by post-hoc Tukey-Kramer HSD multiple comparison.

Of the CpG loci analyzed, methylation at the Alu and SAT2 sequences in non-cancerous liver tissue from HCC patients was slightly lower than that in normal liver (*p* = 0.0494 for Alu and *p* = 0.0334 for SAT2). In HCC tissues, a progressive decrease in methylation at the LINE-1 sequence was correlated with tumor dedifferentiation (*p* = 0.0309 for well-differentiated HCC <2.0 cm vs. >2.0 cm, and *p* = 0.0083 for well-differentiated HCC < 2.0 cm vs. moderately/poorly differentiated HCC; post-hoc Tukey-Kramer HSD multiple comparison). In contrast, Alu and SAT2 sequences did not show significant differences in methylation at different HCC stages.

　‘Normal’ denotes normal liver tissues; ‘NC’ denotes non-cancerous liver; ‘Well’ denotes well-differentiated HCCs; and ‘Mod./poor’ denotes moderately/poorly differentiated HCC. Significant differences (*p* < 0.0001) are shown in bold lines. The F and *p* values for each ANOVA test are as follows: F (4, 374) = 21.87, *p* < 0.0001 for Alu; F (4, 375) = 50.08, *p* < 0.0001 for LINE-1; F (4, 374) = 29.83, *p* < 0.0001 for SAT2.
