# Supplementary material for: Unique Association between Global DNA Hypomethylation and Chromosomal Alterations in Human Hepatocellular Carcinoma
Source: PLoS One. 2013 Sep 2;8(9):e72312. doi: 10.1371/journal.pone.0072312 (PMC3759381; doi:10.1371/journal.pone.0072312)
Supplement: Figure S2 — Hierarchical clustering analysis for categorization of methylation status of global hypomethylation and hypermethylation of TSGs in well-differentiated HCCs and moderately or poorly differentiated HCCs. (DOC) [file pone.0072312.s002.doc]

**Supplementary Figure S2:**

**Hierarchical clustering analysis for categorization of methylation status of global hypomethylation and hypermethylation of TSGs in well-differentiated HCCs and moderately or poorly differentiated HCCs.**

(A) Well-differentiated HCCs were classified either as having “significant” or “slight” global hypomethylation based on the methylation signatures at three different repetitive DNA elements (Alu*,* LINE-1 and SAT2).


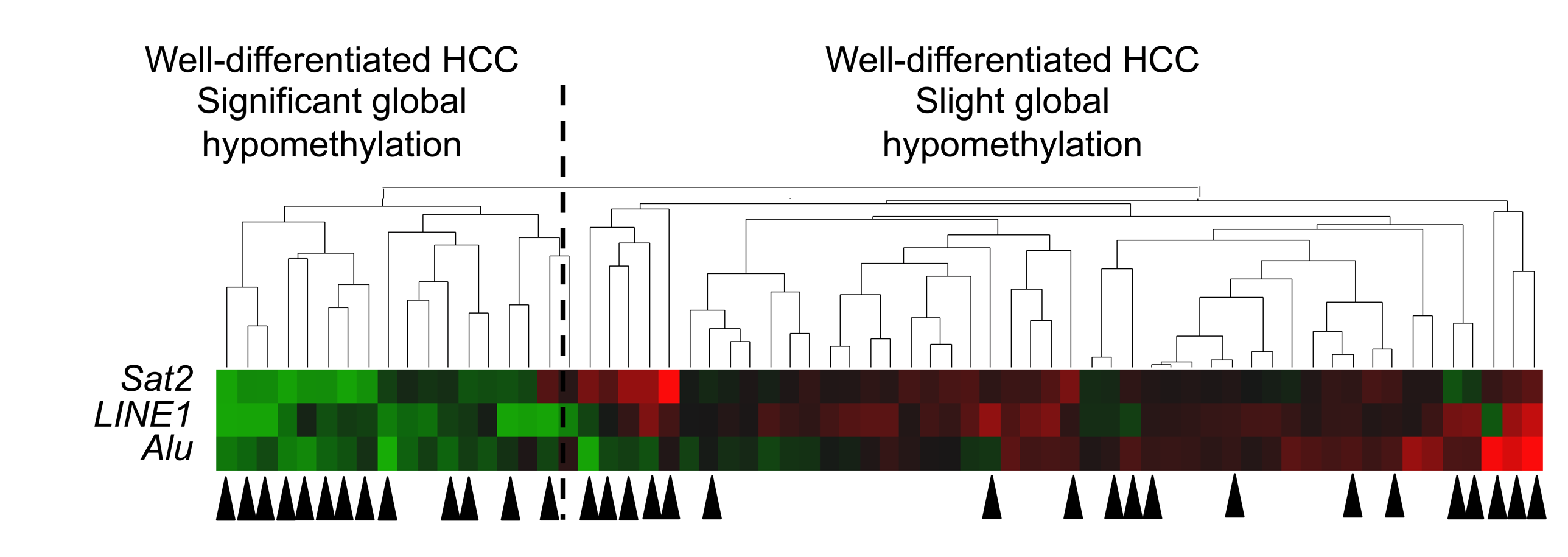


(B) Similarly, well-differentiated HCCs were classified as “limited” or extensive TSG hypermethylation on the methylation signatures at promoters of 11 TSGs (*HIC1, RUNX3, PTGS2, CACNA1G, CDKN2A, PRDM2, CASP8, APC, RASSF1, GSTP1,* and *SOCS1*) and the MINT loci (MINT31).


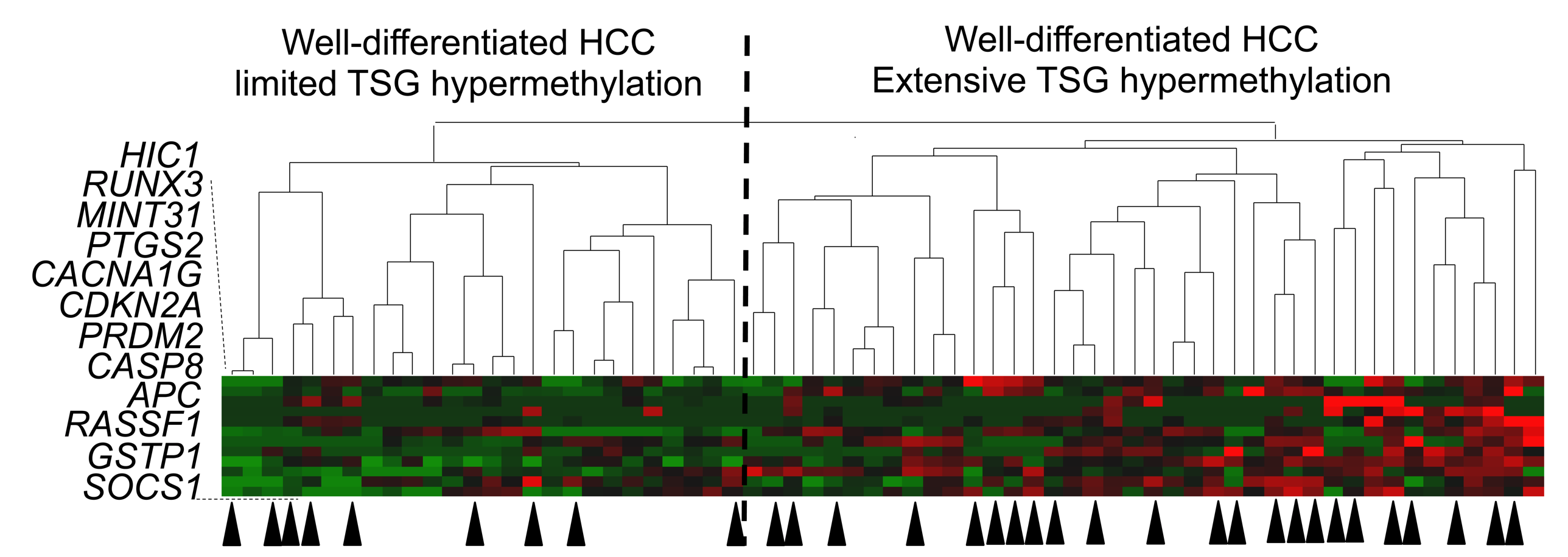


(C) Classification of moderately or poorly–differentiated HCC according to methylation status of three different repetitive DNA elements.


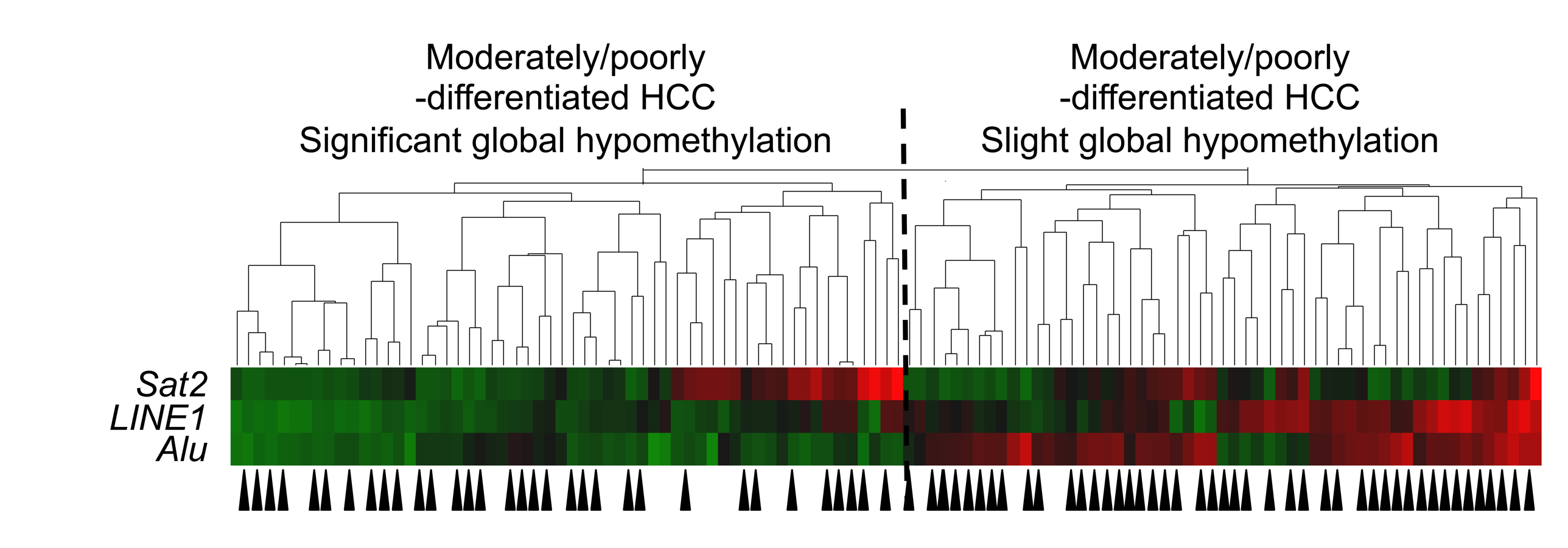


(D) Classification of moderately or poorly–differentiated HCC by hypermethylation of TSGs and a MINT loci.


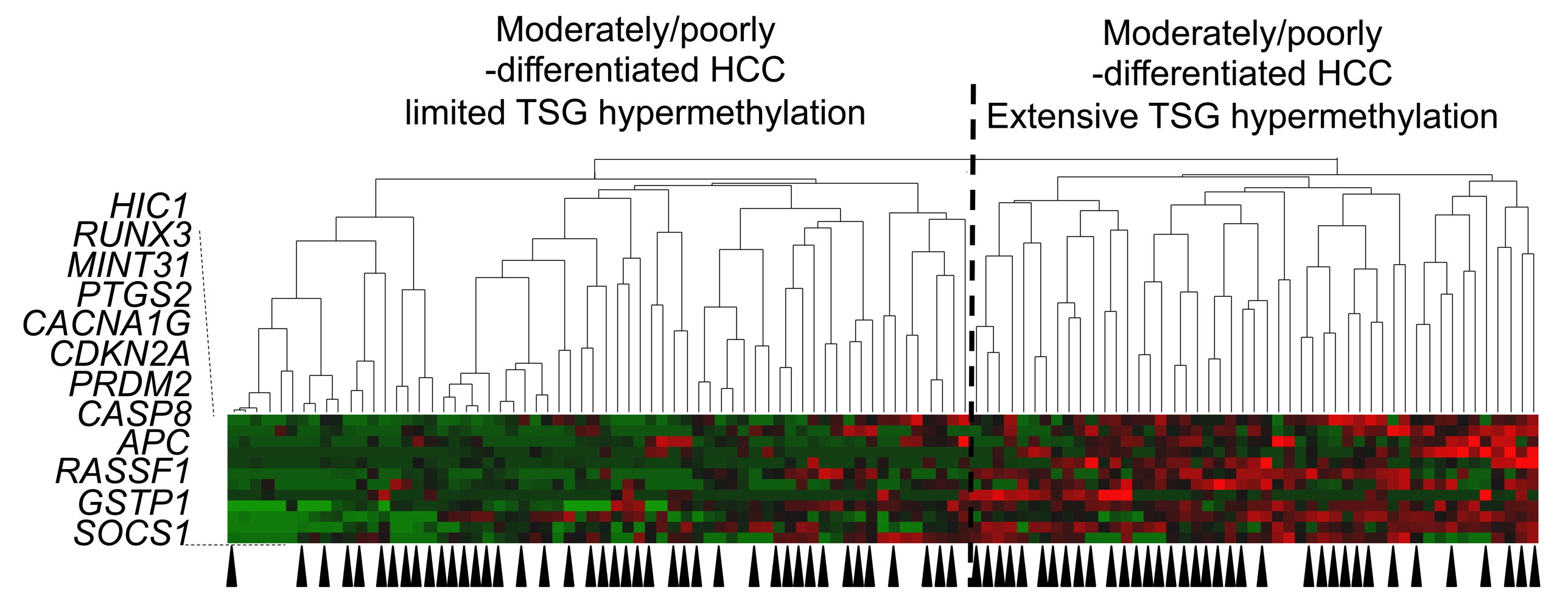


The FAL score was determined for 32 well-differentiated HCCs: 13 were classified as having significant global hypomethylation and 19 as having slight hypomethylation. Similarly, 23 tumors were classified as having extensive TSG hypermethylation and 9 with limited hypermethylation. The FAL score was also calculated for 78 out of 113 moderately or poorly differentiated HCCs. Of these, 33 tumors were classified as having significant global hypomethylation and the remaining 45 as having slight hypomethylation; 36 tumors had extensive TSG hypermethylation and 42 with limited hypermethylation. Arrowheads show the cases whose FAL scores are determined.
