# Supplementary material for: Unique Association between Global DNA Hypomethylation and Chromosomal Alterations in Human Hepatocellular Carcinoma
Source: PLoS One. 2013 Sep 2;8(9):e72312. doi: 10.1371/journal.pone.0072312 (PMC3759381; doi:10.1371/journal.pone.0072312)
Supplement: Table S1 — Difference of methylation levels of Alu, LINE-1, and SAT2 between tumor with significant hypomethylation and with slight hypomethylation. (DOC) [file pone.0072312.s003.doc]

**Supplementary Table S1: Difference of methylation levels of Alu, LINE-1, and SAT2 between tumor with significant hypomethylation and with slight hypomethylation.**

|  |  | Methylation level of tumors | | | *P* value |
| --- | --- | --- | --- | --- | --- |
| CpG loci |  | with significant hypomethylation |  | with slight hypomethylation |  |
| **Alu** |  |  |  |  |  |
| Mean (95% CI) |  | 0.280 (0.253 – 0.308) |  | 0.530 (0.505 – 0.556) | **< 0.0001*** |
| Median (25^th^–75^th^ percentiles) |  | 0.265 (0.219 – 0.351) |  | 0.505 (0.432 – 0.625) | **< 0.0001**† |
|  |  |  |  |  |  |
| **LINE-1** |  |  |  |  |  |
| Mean (95% CI) |  | 0.225 (0.193 – 0.258) |  | 0.494 (0.464 – 0.525) | **< 0.0001*** |
| Median (25^th^–75^th^ percentiles) |  | 0.210 (0.123 – 0.306) |  | 0.487 (0.397 – 0.588) | **< 0.0001**† |
|  |  |  |  |  |  |
| **SAT2** |  |  |  |  |  |
| Mean (95% CI) |  | 0.433 (0.362 – 0.505) |  | 0.504 (0.437 – 0.570) | 0.1158* |
| Median (25^th^–75^th^ percentiles) |  | 0.293 (0.118 – 0.708) |  | 0.537 (0.360 – 0.632) | **0.094**† |

CI, confidence interval; **p* value by Student’s *t*-test; †*p* value by Wilcoxon rank-sum test. Values in bold denote significant differences in methylation levels between tumors with significant hypomethylation and with slight hypomethylation.
