# Supplementary material for: Unique Association between Global DNA Hypomethylation and Chromosomal Alterations in Human Hepatocellular Carcinoma
Source: PLoS One. 2013 Sep 2;8(9):e72312. doi: 10.1371/journal.pone.0072312 (PMC3759381; doi:10.1371/journal.pone.0072312)
Supplement: Table S2 — Univariate analysis of the contribution of each variable to significant hypomethylation at repetitive DNA sequences in HCC. (DOC) [file pone.0072312.s004.doc]

**Supplementary Table S2**

**Univariate analysis of the contribution of each variable to significant hypomethylation at repetitive DNA sequences in HCC**

| Variables |  | Total No. of cases | No. of cases with significant hypomethylation (%) | No. of cases with slight hypomethylation (%) |  | *p* value* |
| --- | --- | --- | --- | --- | --- | --- |
| Age |  |  |  |  |  |  |
| <60 y.o. |  | 96 | 37 (39%) | 59 (61%) |  |  |
| >60 y.o. |  | 80 | 44 (55%) | 36 (45%) |  | **0.0292** |
|  |  |  |  |  |  |  |
| Gender |  |  |  |  |  |  |
| Male |  | 119 | 61 (51%) | 58 (49%) |  |  |
| Female |  | 57 | 20 (35%) | 37 (65%) |  | **0.0440** |
|  |  |  |  |  |  |  |
| Virus status |  |  |  |  |  |  |
| HCV |  | 118 | 48 (49%) | 70 (51%) |  |  |
| Non-HCV |  | 61 | 35 (57%) | 26 (43%) |  | **0.0337** |
|  |  |  |  |  |  |  |
| Normal adjacent liver | | |  |  |  |  |
| LC |  | 116 | 40 (34%) | 76 (66%) |  |  |
| Non-LC |  | 55 | 36 (65%) | 19 (35%) |  | **0.0001** |
|  |  |  |  |  |  |  |
| Serum AFP level |  |  |  |  |  |  |
| < 20 ng/ml |  | 36 | 22 (61%) | 14 (39%) |  |  |
| > 20 ng/ml |  | 54 | 33 (61%) | 21 (39%) |  | 1.0000 |
|  |  |  |  |  |  |  |
| Tumor size |  |  |  |  |  |  |
| < 2.0 cm |  | 42 | 7 (17%) | 34 (83%) |  |  |
| > 2.0 cm |  | 132 | 70 (53%) | 62 (47 %) |  | **< 0.0001** |
|  |  |  |  |  |  |  |
| Differentiation |  |  |  |  |  |  |
| Well |  | 66 | 22 (33%) | 44 (67%) |  |  |
| Moderately or poorly |  | 113 | 61 (54%) | 52 (46%) |  | **0.0075** |
|  |  |  |  |  |  |  |
| FAL score (%) |  |  |  |  |  |  |
| <20 |  | 57 | 20 (35%) | 37 (65%) |  |  |
| >20 |  | 53 | 32 (60%) | 21 (40%) |  | **0.0079** |
|  |  |  |  |  |  |  |
| Mean (95% CI) † |  | – | 27.1 (23.4–30.8) | 18.5 (15.0–22.1) |  | **0.0012** |
| Median (25^th^–75^th^ percentiles) | | – | 23.1 (16.3–38.7) | 17.0 (9.0–24.2) |  | **0.0023** |

CI, confidence interval; HCC, hepatocellular carcinoma; *, *p* value according to the chi-square test for comparison of two categorized variables. For samples with contentious FAL scores, comparison between two tumors with significant and slight hypomethylation was done using Student’s *t*-test and Wilcoxon rank-sum test. †Mean value (95% CI) and median value (25^th^–75^th^ percentiles) are shown. *p* values <0.05, as determined by Student’s *t*-test and Wilcoxon rank-sum test, are shown in bold.
