# Supplementary material for: Unique Association between Global DNA Hypomethylation and Chromosomal Alterations in Human Hepatocellular Carcinoma
Source: PLoS One. 2013 Sep 2;8(9):e72312. doi: 10.1371/journal.pone.0072312 (PMC3759381; doi:10.1371/journal.pone.0072312)
Supplement: Table S3 — Association between progression of hypomethylation and FAL score of HCC. (DOC) [file pone.0072312.s005.doc]

**Supplementary Table S3:**

**Association between progression of hypomethylation and FAL score of HCC.**

| FAL score of tumors |  | Difference of methylation level | | | *P* value |
| --- | --- | --- | --- | --- | --- |
|  |  | With progressive hypomethylation |  | Without progressive hypomethylation |  |
|  |  |  |  |  |  |
| Mean (95% CI) |  | 24.7 (22.7 – 26.6) |  | 19.9 (17.4 – 22.5) | 0.0040* |
| Median (25^th^–75^th^ percentiles) |  | 21.2 (14.0 – 35.5) |  | 18.0 (10.8 – 27.0) | 0.0056† |

Difference of methylation level between HCC and non-cancerous liver was calculated using *Z* score; the relationship between difference of methylation level and FAL score was shown. The median difference of *Z* scores was 0.3. Therefore, we classified cases as with progressive hypomethylation if difference of *Z* score was 0.3 or more. CI, confidence interval; **p* value by Student’s *t*-test; †*p* value by Wilcoxon rank-sum test.
